# 

**Published:** 1952-01

**Authors:** 


### THE BRISTOL MEDICO-CHIRURGICAL JOURNAL

ARMY
MEDICAL
FEB 21 1952
LIBRARY

*January 1952*

VOL. 69. No. 249

